# Heterodimerization of Chemoreceptors TAS1R3 and mGlu_2_ in Human Blood Leukocytes

**DOI:** 10.3390/ijms241612942

**Published:** 2023-08-18

**Authors:** Lena Ball, Julia Bauer, Dietmar Krautwurst

**Affiliations:** 1 TUM School of Life Sciences, Technical University of Munich, Alte Akademie 8a, 85354 Freising, Germany; l.ball.leibniz-lsb@tum.de; 2 Leibniz-Institute for Food Systems Biology at the Technical University of Munich, Lise-Meitner-Str. 34, 85354 Freising, Germany; j.bauer.leibniz-lsb@tum.de

**Keywords:** chemosensory, taste receptors, transcript regulation, immunocytochemistry, BRET, ELISA, fMLF, calcium fluorescence flow cytometry

## Abstract

The expression of canonical chemosensory receptors of the tongue, such as the heteromeric sweet taste (TAS1R2/TAS1R3) and umami taste (TAS1R1/TAS1R3) receptors, has been demonstrated in many extra-oral cells and tissues. Gene expression studies have revealed transcripts for all TAS1 and metabotropic glutamate (mGlu) receptors in different types of immune cells, where they are involved, for example, in the chemotaxis of human neutrophils and the protection of T cells from activation-induced cell death. Like other class-C G protein-coupling receptors (GPCRs), TAS1Rs and mGlu receptors form heteromers within their families. Since mGlu receptors and TAS1R1/TAS1R3 share the same ligand, monosodium glutamate (MSG), we hypothesized their hitherto unknown heteromerization across receptor families in leukocytes. Here we show, by means of immunocytochemistry and co-IP/Western analysis, that across class-C GPCR families, mGlu_2_ and TAS1R3 co-localize and heterodimerize in blood leukocytes. Expressing the recombinant receptors in HEK-293 cells, we validated their heterodimerization by bioluminescence resonance energy transfer. We demonstrate MSG-induced, mGlu_2_/TAS1R3 heteromer-dependent gain-of-function and pertussis toxin-sensitive signaling in luminescence assays. Notably, we show that mGlu_2_/TAS1R3 is necessary and sufficient for MSG-induced facilitation of N-formyl-methionyl-leucyl-phenylalanine-stimulated IL-8 secretion in neutrophils, using receptor-specific antagonists. In summary, our results demonstrate mGlu_2_/TAS1R3 heterodimerization in leukocytes, suggesting cellular function-tailored chemoreceptor combinations to modulate cellular immune responses.

## 1. Introduction

The assembly of GPCRs into homo- and/or hetero-oligomeric complexes has been recognized to modulate their agonist-induced signaling and subcellular trafficking [1,2,3,4]. Indeed, homo- and/or hetero-oligomerization has been reported for ~200 GPCRs [5,6]. Depending on the combination of different receptor subunits in the dimeric complexes, their ligand recognition or sensitivity to stimuli may be modulated. Heterodimerization between the class-C G protein-coupling taste receptors, for example, leads to sweet taste (TA1R2/TAS1R3) or umami taste (TAS1R1/TAS1R3) receptors with effectively different ligand spectra, where none of the monomers can operate independently [7,8,9].

mGluRs, another family of class-C GPCRs, in contrast, were long thought to only exist as homodimeric complexes [10]. Recent studies, however, revealed their assembling into heteromers as well. Heterologous expression analyses showed a physical interaction between group I mGlu receptors (mGlu_1/5_) in the brain [11], as well as functional intra- and intergroup heteromeric receptors within or between groups II (mGlu_2/3_) and III (mGlu_4,6,7,8_) [12,13], in particular between mGlu_2_ and mGlu_4_ [14,15,16] and also between mGlu_2_ and mGlu_7_ [13]. However, the impact of mGlu receptor heterodimerization on their respective cellular functions are still largely obscure.

GPCRs are important sensors at the surface of blood leukocytes, and, upon activation by their adequate stimuli, trigger a variety of cellular immune functions [17,18,19,20]. For example, free fatty acid receptors (FFAR) [21,22], chemokine receptors [23,24], complement receptors [25], and formyl peptide receptors [26,27,28] play fundamental roles in the innate and adaptive immune system, as they detect pathogenic agents, direct cells to the site of injury or infection, and coordinate immune responses.

Moreover, chemosensory GPCRs, such as TAS1- and TAS2-type sweet, umami, and bitter taste receptors from the tongue, have been reported recently also as markers of sub-populations of human blood leukocytes [29,30], responding to and being regulated by food-related compounds, such as non-nutritive sweeteners [29,31]. TAS1 and TAS2 receptors have further been suggested as sentinels of innate immunity in several microbiome-exposed border epithelia, e.g., airway and gastrointestinal epithelia [32,33,34]. Most recently, Qin et al. (2023) proposed that TAS1R3-expressing type II taste cells of the tongue participate in mucosal immune surveillance, based on the observation that type II taste cells have a transcriptome signature reminiscent of microfold cells in mucosa-associated lymphoid tissue [35,36].

The most abundant intracellular amino acid, L-glutamate [37], has a decisive role by functioning as a signaling molecule and immunoregulator via inonotropic and metabotropic glutamate receptors [38]. Gene expression for L-glutamate-detecting G protein-coupling metabotropic glutamate receptors was originally identified in the central nervous system [39,40]. mGlu_1_ and mGlu_4_, however, have been suggested recently to participate in umami detection in the taste buds of the tongue [41]. Furthermore, the expression of mGlu receptors has been demonstrated in different immune cells. Here, they are involved, for example, in the glutamate-induced migration of human polymorphonuclear neutrophils (PMNs) [42,43], in Ca^2+^ signaling and gene expression in human T cells [44], in the protection of T cells from activation-induced cell death (AICD) [45], and in the modulation of adaptive immunity [46]. In addition, group I mGlu receptors (mGlu_1,5_) were shown to mediate opposite effects on T cell proliferation [47,48]. Another study reported that the gene expression for group II mGlu_2_ was markedly reduced in T cells of amyotrophic lateral sclerosis patients [49].

TAS1 and mGlu receptors, both belonging to the class-C GPCRs, are characterized by their large extracellular N-terminal Venus flytrap domain (VFD) that contains the agonist binding site and a cysteine-rich domain (CRD) that connects the VFD with the G protein-activating seven-transmembrane domain [50,51]. A recent study predicted the structure of a fully activated, bound to natural sugars, and G protein-bound TAS1R3 homodimer, showing that the interdomain twisting of the VFDs, which has been associated with receptor activation, is analogous to that of the mGlu receptors [52]. These distinctive features enable class-C GPCRs to assemble into constitutive homo- or heteromeric complexes that are required for G-protein signaling to accomplish their diverse cellular functions [10,13,50,53]. A co-expression or even a heterodimerization, however, of TAS1 and mGlu receptors across class-C GPCR families in blood leukocytes has been unknown, so far.

Here, we set out to investigate the gene expression and their regulation by MSG of all eight mGlu and TAS1 receptors in isolated human blood PMNs and T cells by RT-qPCR. We interrogate the co-expression of mGlu_2_ and TAS1R3 in isolated PMNs by means of immunocytochemistry. We further investigate their heterodimerization in isolated PMNs and T cells by co-immunoprecipitation and Western blotting and validate the heterodimerization of recombinant mGlu_2_ and TAS1R3 in HEK-293 cells by means of bioluminescence resonance energy transfer (BRET). We tested the involvement of mGlu_2_ and TAS1R3 in a functional heterodimerization by cAMP-dependent luminescence assays in HEK-293 cells and by cytokine IL-8 ELISA assays in isolated PMNs, using physiological concentrations of MSG and receptor-specific agonist and antagonists.

## 2. Results

The mRNA expression levels of metabotropic glutamate receptors in human blood leukocytes have been unknown so far. We, therefore, set out to quantify gene transcripts of all *GRMs* and *TAS1Rs* in isolated PMNs and T cells obtained from human buffy-coat samples.

### 2.1. Gene Transcripts of All GRMs and TAS1Rs Are Expressed in Human PMNs and T Cells

**Figure 1:**
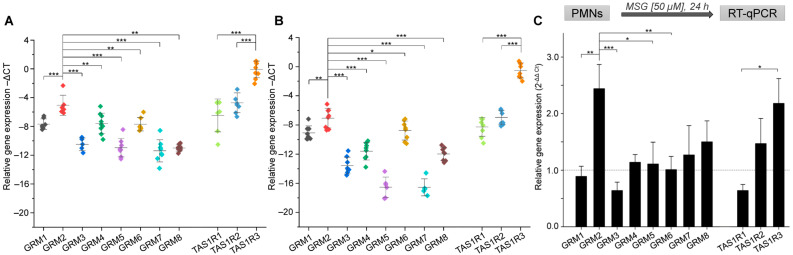
GRM and TAS1R gene transcripts are expressed in human PMNs and T cells. (**A**,**B**) RT-qPCR demonstrates relative quantitative mRNA expression of *GRM* and *TAS1R* genes in human blood PMNs (**A**) and T cells (**B**). Data are shown as mean ± SD (*n* = 8, T cells; *n* = 9, PMNs). (**C**) Relative mRNA expression of *GRMs* and *TAS1Rs* in human PMNs after 24 h MSG stimulation. Data are shown as mean ± SEM (*n* = 8–15). Data were normalized to an averaged expression of two different reference genes (–ΔCT, *GAPDH* + *ACTB*). Transcript levels of *GRM2* were significantly different compared to those of other *GRMs* and between *TAS1R3* and the other *TAS1Rs*, as tested using a two-sided student’s *t*-test: (***) *p* ≤ 0.001; (**) *p* ≤ 0.01; (*) *p* ≤ 0.05. The dotted line indicates the fold change of 1, where there is no change in gene expression levels.

By RT-qPCR, we could confirm the existence of all eight *GRM* as well as of all *TAS1R* gene transcripts in blood leukocytes (Figure 1A,B). Interestingly, within their respective families, *GRM2* and *TAS1R3* revealed the significantly highest gene transcript levels, independent of cell type, underlining the importance of these two receptors in the immune system. Notably, for all *GRMs* and all *TAS1Rs*, except for *TAS1R3*, we observed higher mRNA expression levels in PMNs than in T cells.

For both cell types, *TAS1R3* showed the highest RNA expression among all tested GPCR genes. With ΔCt values around 0, it was expressed as frequently as the housekeeping genes *GAPDH* and *ACTB*. These results are consistent with results from our previous study that revealed TAS1R3 as the receptor with the highest RNA expression among all TAS1R and TAS2R genes in five different types of human blood leukocytes [29]. Moreover, we analyzed the percentage occurrence of all three known isoforms of *GRM2* in PMNs and T cells using ddPCR and identified the longest isoform of *GRM2* (accession number: NM_000839.5) as the isoform with the highest abundance (Figure S1). In both cell types, this transcript represented >80% of *GRM2* isoforms (PMNs: 81%; T cells: 83%).

In the central and peripheral nervous system, the main ligand of metabotropic glutamate receptors is the amino acid L-glutamate [54], which acts as an excitatory neurotransmitter [55]. With regard to taste receptors, L-glutamate is an agonist at least for the umami receptor heteromer TAS1R1/TAS1R3 on the tongue [9]. Since mGlu receptors and TAS1R1/TAS1R3 are sharing the same ligand, we investigated the effect of a 24 h incubation with MSG at a typical plasma concentration of 50 µM [56] on the expression levels of *GRM* and *TAS1* receptor genes in isolated PMNs in vitro. Transcriptional regulation of *GRM2*, for example, in primary neurons has been shown to be significant and persistent up to 24 h and longer in previous studies [57,58]—we did not, however, perform time kinetics for receptor gene expression. Our RT-qPCR-based analysis of mRNA levels revealed a 2.4-fold higher relative gene expression of *GRM2* and a 2.1-fold increase in *TAS1R3* transcripts, significantly different from the transcript levels of other *GRMs* and *TAS1Rs* (Figure 1C).

### 2.2. Immunocytochemistry Revealed Co-Expression of mGlu_2_ and TAS1R3 in Isolated PMNs

**Figure 2:**
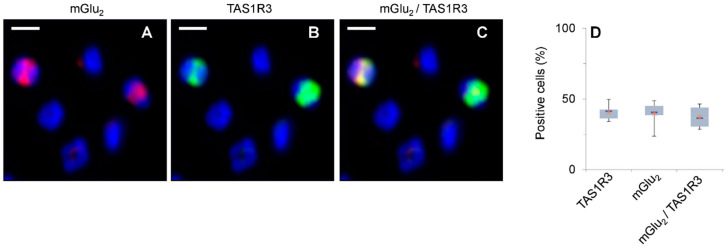
Two-color immunocytochemistry revealed co-expression of mGlu_2_ and TAS1R3 in a sub-population of isolated PMNs. (**A**–**C**) Co-localization of mGlu_2_ and TAS1R3 in isolated PMNs (*n* = 6 blood samples, 3005 cells investigated). (**A**) anti-mGlu2 antibody and secondary antibody carrying fluorophore MFP631 (red); (**B**) anti-TAS1R3 antibody and secondary antibody carrying fluorophore MFP555 (green); (**C**) overlay of the signals in (**A**,**B**) (yellow). Cell nuclei were stained with Hoechst-33342 (blue). Original scale bars, 5 µm. (**D**) Box-whisker plots demonstrate the range of receptor positive cells. Lower and upper grey bars display 2nd and 3rd quartiles of data distribution; horizontal lines, median; X, data means.

Since our gene expression analysis showed the highest transcript levels for *GRM2* and *TAS1R3* in human blood leukocytes, we then asked whether these receptors co-express in the same cells. By using immunocytochemistry, we investigated the protein expression of mGlu_2_ and TAS1R3 in isolated human PMNs in vitro. Notably, 2-color immunocytochemistry revealed that mGlu_2_ and TAS1R3 are co-expressed in a sub-population of approx. 40% of PMNs (Figure 2).

### 2.3. Western Blot and co-IP Revealed Heterodimerization of mGlu_2_ and TAS1R3 in Isolated PMNs and T Cells

**Figure 3:**
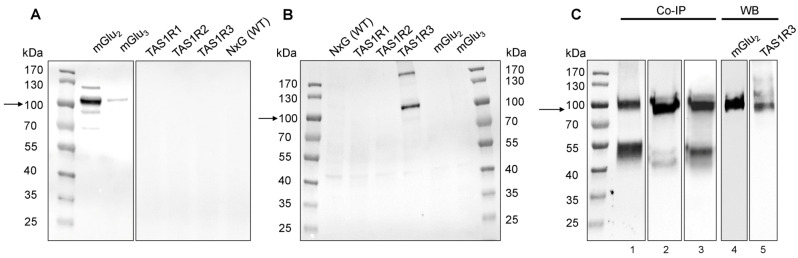
Protein expression of mGlu_2_ and TAS1R3 in human blood PMNs. (**A**,**B**) Antibody validation of anti-mGlu_2_ (**A**) and anti-TAS1R3 antibody (**B**). Specificity of antibodies was tested by transfecting NxG cells with the respective recombinant receptors. Cell extracts were subjected to SDS-PAGE with an amount of 20 µg protein/lane. (**C**) Co-IP assay of mGlu_2_ and TAS1R3 in human blood PMNs. Cell lysates from PMNs (*n* = 3) were incubated with anti-mGlu_2_ (lane 1,) or anti-TAS1R3 antibody (lanes 2 + 3), attached to Dynabeads^®^. Lane 3, same as lane 2, but using 15,000× *g* (instead of 12,000× *g*) to remove membrane and unsolubilized receptor. For Western blot, whole cell lysates and immunoprecipitates were subjected to SDS-PAGE and were analyzed by immunoblotting using anti-TAS1R3 (lanes 1 + 5) or anti-mGlu_2_ antibodies (lanes 2–4). The predicted size of mGlu_2_ protein is ~110 kDa, for TAS1R3 protein ~ 97 kDa, indicated by the black arrows.

Conventionally, coimmunoprecipitation (co-IP) in combination with Western blotting (WB) is used to test protein–protein interactions [59,60]. Due to the lack of receptor-specific antibodies, however, in a first step we performed a validation of our antibodies (Table S3) against mGlu_2_ and TAS1R3. Transfecting NxG cells [61] with the respective receptor plasmids for TAS1R1-3, mGlu_2_, and mGlu_3_ and subsequent WB analysis revealed specificity of the tested antibodies for mGlu_2_ (Figure 3A) and TAS1R3 (Figure 3B). We did, however, also observe a faint band for mGlu_3_, which can be explained by the high sequence identity (67%) of the two group II mGlu receptors, mGlu_2_ and mGlu_3_.

We then investigated using co-IP experiments whether mGlu_2_ and TAS1R3 heterodimerize in isolated PMNs or T cells. Co-IP experiments and subsequent Western blot analysis revealed bands of appropriate sizes (Figure 3C and Figure S2, arrows), indicating a heterodimerization of mGlu_2_ and TAS1R3 in both isolated PMNs (Figure 3C) and T cells (Figure S2). Using 12,000× *g* or 15,000× *g* at the centrifugation step to remove the membrane and any unsolubilized receptors [60] yielded an identical result (Figure 3C, lanes 2 and 3).

### 2.4. Recombinant mGlu_2_ and TAS1R3 Form Heteromeric Complexes in HEK-293 Cells

**Figure 4:**
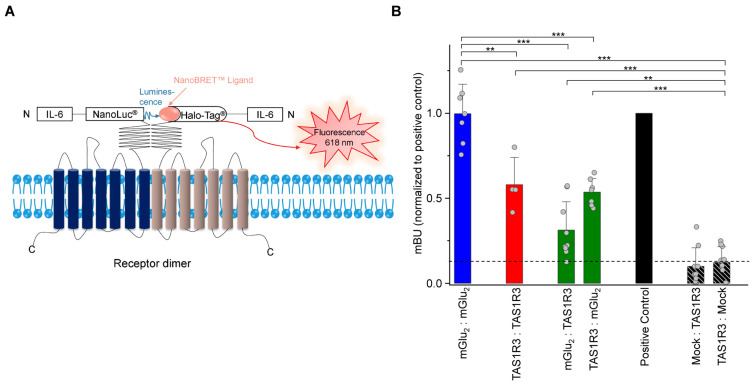
BRET detection of heterodimerization of recombinant mGlu_2_/TAS1R3 in HEK-293 cells. (**A**) Schematic overview of the NanoBRET™ system. Protein–protein interactions, in this case receptor dimerization, result in an energy transfer from a luminescent donor, the IL-6-NanoLuc^®^ luciferase, to the IL-6-HaloTag^®^ binding NanoBRET™ ligand, a fluorescent acceptor, whose excitation can be measured at 618 nm. (**B**) Results of the NanoBRET™-assay. The first mentioned receptor was always expressed out of vector pFN210A carrying the IL-6-HaloTag, the second one out of pNsecNLuc carrying the NanoLuc. Data are presented as the means ± SD of *n* = 4–11 independent experiments, normalized to positive control (PPI p53-pFN:MDM2-NL). Dashed line represents the highest value of the negative controls (black hatched bars). mBU, milli-BRET units. Significance of difference between receptor combinations and mock control was tested using a two-sided student’s *t*-test: (***) *p* ≤ 0.001; (**) *p* ≤ 0.01.

Since we aimed to investigate the effect of a heterodimerization of recombinant mGlu_2_ and TAS1R3 in functional assays in test cells, we first determined a protein–protein interaction of mGlu_2_ and TAS1R3 in living HEK-293 cells by means of bioluminescence resonance energy transfer (BRET), using the NanoBRET™ system [62]. mGlu_2_ and TAS1R3 were labeled with two different N-terminal tags, the IL-6-NanoLuc^®^, which acts as a bioluminescence donor, or the fluorescence acceptor IL-6-HaloTag^®^ (Figure 4A). Receptor interactions were monitored via a luciferase enzyme, with the PPI p53-pFN:MDM2-NL combination as a positive control. We confirmed the specificity of the BRET signal by titrating the fluorescence acceptor IL-6-HaloTag^®^-TAS1R3 while keeping the bioluminescence donor IL-6-NanoLuc^®^-mGlu_2_ constant, which resulted in a hyperbolic saturation curve (Figure S3).

For the respective homodimers of mGlu_2_ or TAS1R3, we observed significantly higher BRET signals than for the mock controls—for mGlu_2_/mGlu_2_ even comparable to the signal intensities of the positive control (Figure 4B). Analysis of the two heterodimeric constellations, mGlu_2_-pFN210A and TAS1R3-pNsecNLuc, and vice versa, revealed dimerization of these two GPCRs in HEK-293 cells, with intensities comparable to that for the TAS1R3 homodimer and significantly different when compared to the mock controls (Figure 4B), confirming the heterodimerization results from the Co-IP/WB and analyses. The presence of the agonist MSG (50 µM) did not alter any BRET signals (Figure S4).

### 2.5. Functional Analysis of Recombinant mGlu_2_ and TAS1R3 Revealed a Heterodimer-Related Gain-of-Function in Response to MSG in HEK-293 Cells

**Figure 5:**
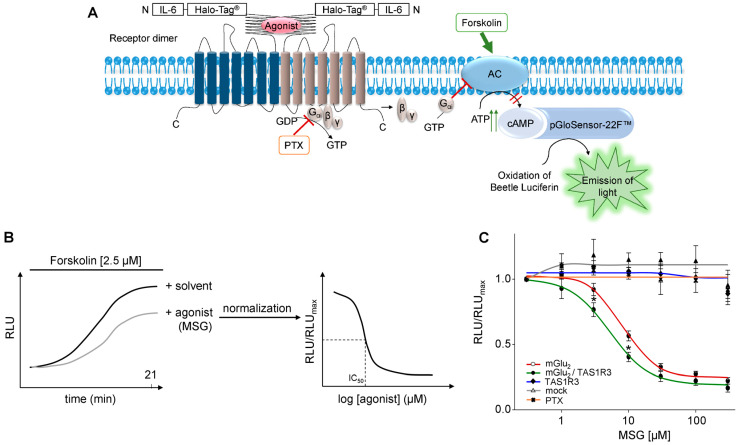
mGlu_2_ and TAS1R3 show gain-of-function in transfected HEK-293 cells. (**A**) Schematic overview of real-time cAMP luminescence-based test cell system. An agonist–receptor interaction results in an activation of the Gα_i_-subunit, which typically inhibits an adenylyl cyclase. A stimulation by forskolin, an adenylyl cyclase activator, leads to an increase in intracellular cAMP, synthesized by an adenylyl cyclase. A simultaneous stimulation of agonist and forskolin leads to an agonist concentration-dependent attenuation of forskolin-induced increase in intracellular cAMP. cAMP binds to a genetically modified luciferase and the emission of light was detected by the GloMax^®^ Discover system (Promega, Madison, WI, USA). (**B**) Experimental procedure. After simultaneous stimulation of the cells with an agonist and forskolin, RLU values were measured for 21 min, until a plateau was detected, where lower RLU values were obtained for the agonist in comparison to the solvent control. Each data set was normalized to its maximum luminescence value leading to a concentration response curve where IC_50_ values were calculated. (**C**) Concentration response curves for MSG in transfected HEK-293 cells. Data are the mean ± SD (*n* = 3, in triplicates). RLU, relative luminescence units. Significance of difference between mGlu_2_ and mGlu_2_/TAS1R3 was tested using the two-sided student’s *t*-test: (*) *p* ≤ 0.05.

A useful tool for a sensitive detection of agonist/receptor interactions in recombinant test cell systems is the GloSensor^TM^ cAMP assay [63,64,65]. In the present study, we had to modify this assay to investigate the MSG-induced function of G_αi_-coupling receptors mGlu_2_ and TAS1R3 (Figure 5A), which supposedly leads to a decrease in cAMP [66]. Therefore, we simultaneously stimulated mGlu_2_- and/or TAS1R3-transfected HEK-293 cells with different concentrations of MSG and forskolin, an diterpene activator of adenylyl cyclase [67,68], and analyzed the concentration/response–relationships of MSG-activated attenuation of forskolin-induced intracellular cAMP accumulation (Figure 5B,C) [66]. We performed these analyses in the presence of different G_αi_ proteins (G_αi1–3_ and G_αGustducin_) and in the absence and presence of pertussis toxin (PTX) [69,70]. We calculated the respective IC_50_ values for both the mGlu_2_ or TAS1R3-transfected HEK-293 cells and the potential mGlu_2_/TAS1R3 heterodimeric transfectants (Figure 5C, Table 1). Notably, TAS1R3-transfected HEK-293 cells did not respond to MSG with a concentration-dependent decrease in cAMP (Figure 5C).

For all G_αi_ proteins tested, we observed significantly lower IC_50_ values for the heterodimer mGlu_2_/TAS1R3 than for the homomer mGlu_2_/mGlu_2_, with G_αi3_ being the most efficient signal transducing G-protein (Table 1). The IC_50_ values for mGlu_2_ are consistent with values from a previous study, which reported half-maximal inhibitory concentrations of 4–20 µM L-glutamate [39], confirming the sensitivity of our assay. The concentration–response curve in mGlu_2_/TAS1R3-co-transfected cells is shifted to the left, resulting in a significant 2–3-fold higher sensitivity towards MSG as compared to the mGlu_2_-transfectants, suggesting a heterodimer-dependent gain-of-function, despite the potential presence of homomeric mGlu_2_/mGlu_2_ (Figure 5C).

We additionally evaluated the effect of PTX, which inhibits the interaction of GPCRs with their cognate G_i/o_ proteins [69,70,71]. PTX treatment of mGlu_2_- and/or TAS1R3-transfected HEK-293 cells before stimulation with MSG and forskolin resulted in a complete loss of the MSG concentration-dependent decrease in cAMP (orange curve, Figure 5C).

We then investigated whether the gain-of-function observed with mGlu_2_/TAS1R3-co-transfected cells can also be observed in cells co-transfected with mGlu_2_ and the other members of the TAS1R family, TAS1R1 or TAS1R2. The IC_50_ values obtained revealed no such effect (Table 2), suggesting a mGlu_2_/TAS1R3 heteromer-specific gain-of-function in our luminescence assays with transfected HEK-293 cells.

We further evaluated the HEK-293 cell surface expression of IL-6-HT-mGlu_2_ and IL-6-HT-TAS1R3 receptors using flow cytometry. Interestingly, we observed a significant higher surface expression of the IL-6-HT-mGlu_2_/IL-6-HT-TAS1R3 heteromer as compared to IL-6-HT-mGlu_2_ alone (Figure S5).

Our results, so far, demonstrate that mGlu_2_ and TAS1R3, indeed, heterodimerized in primary PMNs and T cells, and in HEK-293 cells. Here, mGlu_2_/TAS1R3 appeared to be at least necessary for the observed MSG-induced gain-of-function in attenuating a receptor/G protein-mediated decrease in cAMP signaling in luminescence assays. To further elucidate the role of the heteromer mGlu_2_/TAS1R3 in primary immune cells, we used the mGlu_2_- and TAS1R3-specific antagonist on N-formyl-methionyl-leucyl-phenylalanine (fMLF)-induced IL-8 secretion, a typical cellular immune response in neutrophils.

### 2.6. MSG via mGlu_2_/TAS1R3 Facilitated an fMLF-Induced IL-8 Secretion in Isolated PMNs In Vitro

Human neutrophils are the first line of defense in our blood system, where they function as specific sensors for pathogen- or damage-associated molecular patterns (PAMPs or DAMPs), followed by the migration to the site of inflammation [72]. They are activated by chemotactic stimuli such as fMLF, which bind to its cognate receptor, FPR1, leading to, for example, the secretion of cytokines, such as the most potent neutrophil-stimulating chemokine, the CXCL8 (IL-8) [73].

**Figure 6:**
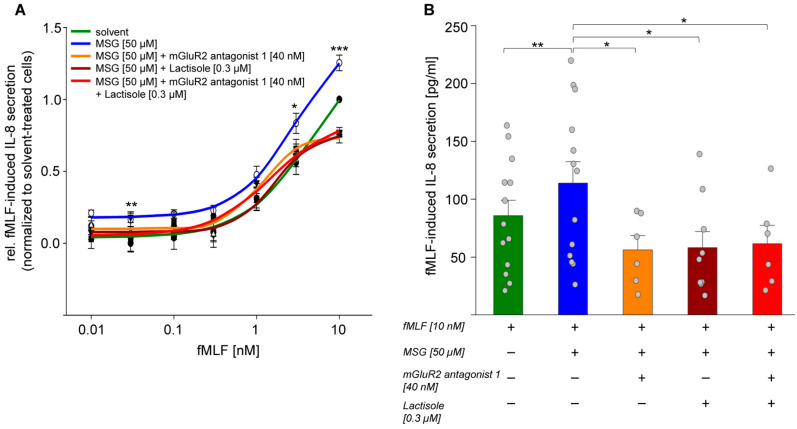
MSG via heteromeric mGlu_2_/TAS1R3 facilitated an fMLF-induced IL-8 secretion in isolated PMNs in vitro. (**A**) 4 h fMLF-induced IL-8 secretion in isolated PMNs, after 2 h pre-stimulation with 50 µM MSG or RPMI-treatment, in the absence or presence of mGlu_2_-specific antagonist 1 and/or TAS1R3-specific antagonist Lactisole. Changes of IL-8 concentration are normalized to RPMI-treated samples without MSG (“solvent”, *n* = 6–13). Data are shown as mean ± SEM. Significance of difference between solvent and MSG pre-stimulated cells was tested using a two-sided student’s *t*-test: (***) *p* ≤ 0.001; (**) *p* ≤ 0.01; (*) *p* ≤ 0.05. (**B**) Maximum fMLF-induced IL-8 concentrations in pg/mL in presence and absence of MSG pre-stimulus or the respective receptor-specific antagonists. Data are the mean ± SEM (*n* = 6–13). Significance of difference was tested using the one-sided student’s *t*-test: (**) *p* ≤ 0.01; (*) *p* ≤ 0.05.

We measured concentration-response relations of fMLF-induced IL-8 secretion by ELISA in PMNs pre-stimulated with a physiological concentration of MSG (50 µM) [56], in the absence or presence of receptor-specific antagonists (Figure 6A). While the EC_50_ values were not significantly different (Table S6), the concentration–response relation of fMLF-induced IL-8 secretion was, however, significantly shifted to a higher IL-8 secretion when PMNs were pre-stimulated with MSG, both at lowest and highest fMLF concentrations (Figure 6A). In cells that were not MSG-pre-stimulated (RPMI 1640-treated), we observed maximum (10 nM) fMLF-induced IL-8 levels of 85.71 ± 46.39 pg/mL. Pre-stimulation with MSG increased the IL-8 concentration up to 113.95 ± 63.99 pg/mL. This effect was completely reversed in the presence of the receptor antagonists. Here, the IL-8 concentrations amounted to 56.15 ± 27.67 pg/mL in the presence of 40 nM mGluR_2_ antagonist 1, 58.14 ± 39.34 pg/mL in the presence of 0.3 µM TAS1R3-specific antagonist Lactisole, and to 61.61 ± 35.51 pg/mL in the presence of both antagonists (Figure 6B). At these concentrations, we did not observe any stimulatory or inhibitory effect of the two antagonists on the concentration–response relationship of fMLF-induced IL-8 secretion (Figure S6). Notably, just the presence of the sweet taste antagonist and TAS1R3-specific antagonist Lactisole completely reverted the MSG-facilitated concentration–response relations of fMLF-induced IL-8 secretion back to the non-facilitated fMLF curve (Figure 6A), suggesting that just a potential mGlu_2_/mGlu_2_ homodimer in PMNs is not sufficient to explain the MSG-dependent facilitation of fMLF-induced IL-8 secretion but rather Lactisole’s effect on a mGlu_2_/TAS1R3 heteromer. Likewise, the mGlu_2_-specific antagonist 1 completely reverted the MSG-facilitated concentration–response relations of fMLF-induced IL-8 secretion back to the non-facilitated fMLF curve (Figure 6A), suggesting that a potential umami receptor TAS1R1/TAS1R3 heteromer in PMNs is likely not involved in the MSG-dependent facilitation of fMLF-induced IL-8 secretion. Altogether, these results suggest that the observed MSG treatment-derived gain of fMLF-induced IL-8 function in isolated primary PMNs depended on the presence of the mGlu_2_/TAS1R3 heteromer.

The mGlu_2_/mGlu_3_-receptor-selective agonist LY379268 at nanomolar concentrations supposedly is selective for mGlu_2_ over mGlu_3_ [74,75]. LY379268, like MSG, significantly facilitated an fMLF-induced IL-8 secretion, which was completely reverted by the mGluR2 antagonist 1 (Figure S7). Altogether, this suggests at least that an activation of specifically the mGlu_2_-receptor is causative for an MSG/LY379268-dependent increase in fMLF-induced IL-8 secretion in isolated PMNs (Figure 6). Lactisole at 0.3 µM did not revert the effect of potent agonist LY379268. We refrained, however, from using higher concentrations, since Lactisole has been shown previously to activate isolated PMNs at concentrations higher than 0.3 µM [29].

### 2.7. MSG and LY379268 Increased Intracellular Ca^2+^ in Isolated PMNs

Intracellular calcium signaling is an important mechanism for a wide range of immune functions and priming in neutrophils [76,77,78]. Following GPCR activation and subsequent modulation of Ca^2+^-store- or ligand-operated Ca^2+^ channels, mediated by various priming agents [31,77,79], there is an increase in neutrophils’ intracellular calcium concentration due to its release from intracellular stores and/or influx via the plasma membrane [76,80,81,82,83].

**Figure 7:**
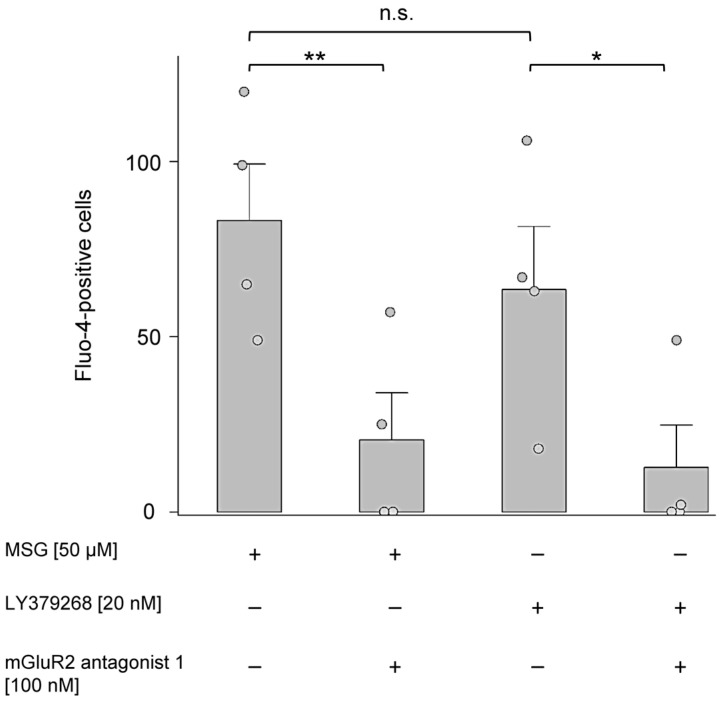
MSG and the mGlu_2_-receptor agonist LY379268 increased intracellular Ca^2+^ in isolated PMNs. Shown are the number of Fluo-4-positive cells after a 2 h incubation with MSG or the mGlu_2_ receptor agonist LY379268 in the presence or absence of mGluR2 antagonist 1. Solvent control (0.1% DMSO) was subtracted from each measurement. Data are expressed as mean ± SEM (*n* = 4). The significance of differences was tested using the one-sided student’s *t*-test: (**) *p* ≤ 0.01; (*) *p* ≤ 0.05, n.s., not significant.

Here, we set out to investigate whether the use of MSG or nanomolar concentration of the mGlu_2_ receptor agonist, LY379268 would influence Ca^2+^ homeostasis in isolated neutrophils. Using the same 2 h stimulation approach as in the IL-8 secretion experiments, we then measured Ca^2+^ fluorescence in Fluo-4-loaded PMNs using flow cytometry. We observed a similar and significantly higher number of Fluo-4-positive cells for MSG and LY379268 as compared to cells stimulated in the presence of mGluR2 antagonist 1 (Figure 7).

## 3. Discussion

The concept of heterodimerization across different families of class C GPCRs in primary human blood leukocytes is new. So far, there has been only one report on heterodimerization of mGlu_2*α*_ with the calcium sensing (CaS) receptor in rat brain but with yet unknown function [84]. In the present study, we demonstrate mRNA expression of all eight *GRMs* and all *TAS1Rs* with distinct but significant abundance in two different primary human blood cell types, PMNs and T cells: in both cell types, *GRM2* and *TAS1R3* consistently showed the highest levels of expression, which for the latter is concordant with our previous study on TAS1Rs in blood leukocytes [29].

The regulation of GPCR gene expression in blood leukocytes is well described [85,86,87]. The regulation of taste receptor expression in extra-oral tissues by their cognate ligands has been demonstrate recently in blood leukocytes [31,88] and in heart [89]. Surprisingly, upon stimulation with MSG, PMNs showed an increased gene expression not only of *GRM2* but also of *TAS1R3*. Both transcript levels were significantly higher compared to the other *GRM* and *TAS1R* genes from the same receptor families.

Despite the most efficient homodimerization of the recombinant IL-6-HT-mGlu_2_/IL-6-HT-mGlu_2_ homomer, as determined by BRET, in our hands, the IL-6-HT-mGlu_2_/IL-6-HT-TAS1R3 heteromer showed a significant higher HEK-293 cell surface expression as compared to the IL-6-HT-mGlu_2_/IL-6-HT-mGlu_2_ homomer. This was corroborated, however, by our functional cAMP luminescence assays, revealing a significant IL-6-HT-mGlu_2_/IL-6-HT-TAS1R3 heteromer-dependent and MSG-induced gain-of-function in attenuating forskolin-activated cAMP signaling in HEK-293 cells, as compared to the IL-6-HT-mGlu_2_/IL-6-HT-mGlu_2_ homomer. Altogether, this may suggest a chaperone function of TAS1R3 as the causative mechanism underlying the observed MSG-induced gain-of-function of the mGlu_2_/TAS1R3 heteromer. Indeed, a C-terminal, membrane-distal, di-basic amino acid motif supposedly functions as intracellular retention signal in TAS1R2, which has been suggested to be masked or dislocated upon interaction with TAS1R3 [90], promoting cell surface expression of the heteromer. A similar mechanism has been demonstrated for another heterodimerizing pair of class-C GPCRs, the GABA_B1_ and GABA_B2_ receptors [91,92].

Several lines of evidence in the present study suggest an assembly of mGlu_2_ and TAS1R3 into a functional heterodimer: (i) both mGlu_2_ and TAS1R3 were co-upregulated in MSG-stimulated PMNs; (ii) mGlu_2_ and TAS1R3 receptors are co-expressed in a subpopulation of PMNs; (iii) co-immunoprecipitation of mGlu_2_ and TAS1R3 PMNs and T cells; (iv) validation of the recombinant mGlu_2_/TAS1R3 heterodimer in HEK-293 cells by BRET; (v) a mGlu_2_/TAS1R3 heteromer-specific gain-of-function in attenuating cAMP signaling in HEK-293; (vi) and an MSG-induced and mGlu_2_/TAS1R3 heteromer-dependent gain of fMLF-stimulated IL-8 function in isolated PMNs.

Is there, however, a role of potential homomeric TAS1R3/TAS1R3 or mGlu_2_/mGlu_2_ receptors or the umami taste TAS1R1/TAS1R3 receptor heteromer in mediating MSG-induced effects in isolated PMNs?

A sweetener-induced and TAS1R3/TAS1R3 homomer-mediated cAMP signaling has been suggested previously [93]. Recently, we reported a Lactisole concentration-dependent migration of isolated neutrophils in vitro, with a significantly increased EC_50_ in TAS1R3-siRNA-treated cells but not in TAS1R2-siRNA-treated cells, suggesting a TAS1R3/TAS1R3 homomer-mediated function in response to its cognate ligand Lactisole [29].

With regard to MSG, however, test cells expressing only recombinant TAS1R3 did not respond to MSG in our hands. This is corroborated by a previous study [8]. Therefore, a potential TAS1R3/TAS1R3 homomer very likely did not mediate any MSG-induced effects in PMNs. However, the TAS1R3-specific sweet taste antagonist Lactisole in the present study completely blocked the MSG facilitation of fMLF-induced IL-8 secretion in PMNs, suggesting (i) that Lactisole exerted its blocking effect via the TAS1R3 subunit of a mGlu2/TAS1R3 heteromer and, thus, (ii) that a potential mGlu_2_/mGlu_2_ homodimer in PMNs is not necessary for the MSG-dependent facilitation of fMLF-induced IL-8 secretion. Since the mGlu_2_ antagonist 1 completely blocked the MSG facilitation of fMLF-induced IL-8 secretion in PMNs, a potential umami receptor TAS1R1/TAS1R3 heteromer appears not involved, either. Moreover, the recombinant umami receptor TAS1R1/TAS1R3 heteromer required MSG concentrations way beyond 50 µM to become activated [94].

The production and release of IL-8 in human neutrophils is Ca^2+^-dependent [78]. Our findings that both MSG and LY379268 increased an intracellular Ca^2+^ fluorescence in isolated PMNs, which was blocked by the mGlu_2_ antagonist 1, may suggest the involvement of mainly the mGlu_2_ receptor as one causative mechanism for an MSG-dependent facilitation of fMLF-induced IL-8 secretion and argues against an involvement of other metabotropic glutamate receptors.

However, our results do not rule out the presence/co-existence of any mGlu receptor homo- or heteromers or mGlu receptor/TAS1R3 heteromers in PMNs. Further experiments, e.g., siRNA-guided, receptor-specific, knock-down experiments, are needed to strengthen a functional role of a mGlu_2_ receptor/TAS1R3 heteromer in PMNs.

Concluding, we infer that the mGlu_2_/TAS1R3 heteromer likely mediated the MSG facilitation of fMLF-induced IL-8 secretion in PMNs.

This facilitation, however, suggests crosstalk between the MSG-activated mGlu_2_/TAS1R3 heteromer and the fMLF-activated FPR1 receptor and their signaling pathways in PMNs. In a most recent study, we reported the facilitation of fMLF/FPR1-induced Ca^2+^ signaling in PMNs by pre-activating non-nutritive sweetener–cognate GPCRs [31]. GPCR crosstalk, heterologous sensitization, and facilitation of fMLF/FPR1-induced Ca^2+^ signaling are well-known principles in PMNs [95,96,97] and, therefore, may be causative mechanisms underlying the MSG facilitation of fMLF-induced IL-8 secretion in PMNs observed in the present study. Thus, we may hypothesize that the simultaneous presence of (i) MSG or other endogenous or microbiome-derived stimuli and metabolites and (ii) pathogen- or damage-associated molecular patterns (PAMPs or DAMPs), such as fMLF, in general may facilitate or modulate cellular immune responses. This notion is corroborated by a recent study, demonstrating that a full activation of PMNs required the simultaneous presence of adequate stimuli for at least two different receptor systems [98].

Recent studies demonstrated that the mGlu_2_ but not the mGlu_3_ receptor lacks both rapid glutamate-induced, GRK-mediated desensitization and arrestin-mediated internalization [99,100]. Moreover, in the present study, we demonstrated that the cell surface expression of the mGlu_2_ receptor increased significantly in the presence of TAS1R3, compared to mGlu_2_ alone, suggesting a chaperone function of TAS1R3. Altogether, these features make the mGlu_2_/TAS1R3 heteromer well suited to function as a constitutive sensor on PMNs or T cells for monitoring, e.g., food ingredients/additives or endogenous or microbiome-derived metabolites, such as MSG, in the blood system or border epithelia. Thus, the functional role of the mGlu_2_/TAS1R3 heteromer in our cellular immune system remains to be investigated by future experiments.

In summary, we demonstrate the differential mRNA expression of all *GRM* and *TAS1R* genes in PMNs and in T cells, with mGlu_2_ and TAS1R3 consistently displaying the highest gene expression levels, which are further increased by stimulation with their cognate agonist MSG. Our results unambiguously demonstrate heterodimerization of both receptors in primary PMNs and T cells, validated by an MSG-induced gain-of-function of recombinant mGlu_2_/TAS1R3 in HEK-293 cells. Moreover, we show that a functional mGlu_2_/TAS1R3 heteromer is necessary and sufficient to explain an MSG-induced facilitation of fMLF-activated IL-8 function in isolated PMNs. Our results demonstrate a heterodimerization across different families of class-C GPCRs in leukocytes, suggesting previously unnoticed, new cellular function-tailored chemoreceptor combinations, enabling our immune system to cope with a vast variety of stimuli.

## 4. Materials and Methods

### 4.1. Chemicals

The following chemicals were used: Dulbecco’s MEM, sodium chloride, 2-Mercaptoethanol (Merck KGaA, Darmstadt, Germany), FBS superior, L-glutamine, Penicillin (10,000 U/mL)/streptomycin (10,000 U/mL), Trypsin/EDTA solution (Bio & Sell GmbH, Feucht, Germany), Dimethyl sulfoxide (DMSO), HEPES, Potassium chloride, sodium hydroxide, calcium chloride dehydrate, D-glucose (VWR International GmbH, Darmstadt, Germany), D-luciferin (beetle) monosodium salt (Promega, Madison, WI, USA), Bordetella pertussis toxin, L-Glutamic acid monosodium salt monohydrate (Santa Cruz Biotechnology, Dallas, TX, USA), Forskolin (Biomol GmbH, Hamburg, Germany), Lactisole (Cayman Chemicals, Ann Arbor, MI, USA), fMLF (Tokio Chemical Industry, Tokyo, Japan), mGluR2 antagonist 1 (CAS: 1432728-49-8; Biozol GmbH, Eching, Germany), metabotropic glutamate receptor 2/3 agonist LY379268 (Tocris Bioscience, Bristiol, UK), Gibco RPMI 1640 containing L-glutamine (Thermo Fisher Scientific, Waltham, MA, USA), Probenecid (Sigma-Aldrich, St. Louis, MO, USA), Pluronic^®^ F-127 (AAT Bioquest Inc., Sunnyvale, CA, USA), Fluo-4 AM (Bio-Techne, Minneapolis, MN, USA). Calcium buffer was composed of 140 mM NaCl, 20 mM HEPES, 5 mM KCl, 1.8 mM CaCl_2_, and 0.5 mM D-glucose, adjusted pH 7.4.

### 4.2. Human Blood Cell Purification

Human blood neutrophils and T cells were isolated from buffy coat samples (Health Center Dr. Becker, Munich, Germany) and purified using MACSxpress^®^ Whole Blood Neutrophil Isolation Kit or Pan T Cell Isolation Kit, human, respectively (Miltenyi Biotec, Bergisch Gladbach, Germany), following the manufacturer’s recommendations. Using the MACSxpress^®^ Erythrocyte Depletion Kit (Miltenyi Biotec), remaining erythrocytes were removed by magnetic depletion.

After isolation, cells were centrifuged at 300 rpm for 10 min and the pellets were stored at −80 °C for RNA isolation. For the stimulation experiments, human neutrophils and T cells were resuspended in RPMI 1640, including 50 µM MSG, and were incubated for 24 h at 37 °C and 5% CO_2_.

### 4.3. RNA Isolation and cDNA Synthesis

The total RNA from human blood neutrophils and T cells was isolated and purified using the RNeasy^®^ Mini Kit with two on-column DNase-I digestions (Qiagen, Hilden, Germany) according to the manufacturer’s recommendations. The concentration of RNA was determined by a NanoDrop™ One spectrophotometer (Thermo Fisher Scientific, Waltham, MA, USA). The cDNA was synthesized from total RNA using the High-Capacity cDNA Reverse Transcription Kit (Thermo Fisher Scientific).

### 4.4. RT-PCR and qPCR

The expression of all *GRM* and *TAS1R* genes in T cells and PMNs was analyzed by RT-qPCR, using gene specific primers (Table S1) and normalized to an average of two stably expressed reference genes *GAPDH* (Glyceraldehyde 3-phosphate dehydrogenase) and *ACTB* (Actin Beta), according to the “Minimum Information for Publication of Quantitative Real-Time PCR Experiments Guidelines” [101] that have been selected previously by geNorm and NormFinder [29,102]. qPCR reactions were performed in duplicates using the 2× SsoAdvanced™ Universal SYBR^®^ Green Supermix (Bio-Rad Laboratories, Hercules, CA, USA) in a CFX96 Touch Real-Time PCR Detection System (Bio-Rad Laboratories, Hercules, CA, USA): 95 °C (1 min), 45 × [95 °C (15 s), 58 °C (60 s)]. Following a slow heating step from 60 °C to 95 °C with a dwell time of 5 s, increments of 0.5 °C, a melting analysis was performed. Ct data and melting curves were evaluated using the CFX Maestro software v.1.1 (Bio-Rad Laboratories, Hercules, CA, USA), incorporating a Ct cut-off ≥ 40. Samples with irregular melting curves were excluded from the quantification analysis. Only genes that showed a clear melting peak were used for further analysis.

### 4.5. ddPCR

ddPCR reactions were carried out in duplicates using the 2× supermix for probes (no dUTP) (Bio-Rad Laboratories, Hercules, CA, USA) following the manufacturer’s instructions of the QX200 Droplet Digital PCR System (Bio-Rad Laboratories, Hercules, CA, USA). Briefly, 100 ng of PMN or T cell cDNA were mixed with primers and probe (final concentrations of 900 nM and 250 nM, respectively) (Table S2) together with the ddPCR supermix in a final volume of 20 µL. For the generation of droplets, the samples were transferred to a DG8 Cartridge (Bio-Rad Laboratories, Hercules, CA, USA). After adding Droplet Generation Oil for Probes, the Cartridge was placed into the QX200 Droplet Generator™ (Bio-Rad Laboratories, Hercules, CA, USA). The droplets were transferred to a semi-skirted 96-well PCR plate which was then immediately heat sealed in the PX1 PCR Plate Sealer (Bio-Rad Laboratories, Hercules, CA, USA) (5 s at 180 °C). The amplification was performed in a 96-well thermal cycler (peqlab, Erlangen, Germany) using the following cycling protocol: 95 °C for 10/min (DNA polymerase activation), 40 × [94 °C for 30/s (denaturation), 58 °C for 1/min (annealing)] and a subsequent enzyme deactivation step at 98 °C for 10 min. The reaction was kept at 4 °C. Droplets were measured in a QX200 Droplet reader (Bio-Rad Laboratories, Hercules, CA, USA) and the data were analyzed using the Quantasoft Version 1.7.4 (Bio-Rad Laboratories, Hercules, USA). Each run included a non-template control. Since *GRM2* isoform 3 (NM_000839.5) does not have a specific sequence pattern, the primer and probe for this isoform were designed to recognize all three isoforms. To determine the percentage occurrence of the respective isoforms, isoforms 1 and 2 were calculated relative to the third isoform, and their common percentages were then subtracted from isoform 3 (Figure S1).

### 4.6. Immunocytochemistry

The protein expression of mGlu_2_ and TAS1R3 in human blood PMNs was evaluated by immunocytochemistry, using receptor-specific antibodies. For TAS1R3, cells were incubated with the rabbit anti-human TAS1R3-specific primary antibody (1:250) (LS-A5060, Biozol GmbH, Eching, Germany) for 24 h at 4 °C, following an application of the secondary goat anti-rabbit MFP555 antibody (1:500) (MFP-A2429, 560/585 nm, excitation/emission, Mobitec GmBH, Goettingen, Germany) for 2 h. For mGlu_2_, a goat anti-human mGlu_2_ antibody (1:200) (LS-B8336, Biozol GmbH, Eching, Germany) was applied for 24 h at 4 °C, following an incubation with the secondary rabbit anti-goat MFP631 antibody (1:500) (MFP-A2086, 633/658 nm, excitation/emission, Mobitec GmBH, Goettingen, Germany) for 2 h. The nuclei of cells were stained with Hoechst-33342 (Invitrogen; 350/461 nm, excitation/emission). Fixation of cells, antibody-staining, and recoding of fluorescence signals were performed as described previously [29], using laser-scanning microscopy (40×, Olympus IX81; Olympus, Melville, NY, USA).

### 4.7. Bioluminescence Resonance Energy Transfer (BRET) Assay

For the BRET assay, we used HEK-293 cells [103] that were cultivated as previously described [65]. Briefly, one day before transfection, cells were cultured in a 96-well format (Thermo Scientific™ Nunc™ F96 MicroWell™, Thermo Fisher Scientific Inc., Waltham, MA, USA) with a density of 12,000 cells per well. Using the ViaFect™ Transfection Reagent (Promega, Madison, WI, USA), 50 ng/well plasmid DNA in the pFN210A vector, 50 ng/well plasmid DNA in the pN[secNluc/MCS/CMV/Neo] Vector, 50 ng/well G protein subunit Gα_i3_, and the cAMP-luciferase pGloSensor™-22F [63] (Promega, Madison, WI, USA) were transiently transfected. For the NanoBRET™PPI Control Pair (p53, MDM2; #N1641, Promega, Madison, WI, USA), each 50 ng/well served as a positive control; as negative controls, a combination of the vector plasmid pN[secNluc/MCS/CMV/Neo] Vector (Kan) (Promega, Madison, WI, USA) lacking any receptor coding region/TAS1R3 (1:1) and the vector plasmid pFN210A lacking any receptor coding region/TAS1R3 (1:1), 50 ng/well each, were used. After incubating the cells for 18–24 h, the NanoBRET Ligand™618 (Promega, Madison, WI, USA) in serum-free medium without phenol red was applied (final concentration on the cells 100 nM). DMSO in serum-free medium without phenol red was used as a no-acceptor control with a final concentration of 0.1% on the cells.

At 42 h post transfection, the cells were washed with serum-free medium without phenol red following the application of the NanoBRET™NanoGlo Substrate (Promega, Madison, WI, USA). Using the GloMax^®^ Discover Microplate Reader (Promega, Madison, WI, USA), the donor emission (460 nm) and the acceptor emission (618 nm) were measured. The NanoBRET™ ration value in milliBRET units (mBU) was obtained by dividing the acceptor emission value by the donor emission value and multiplying by 1000.

### 4.8. Western Blot and Co-IP

For Western blot analysis, NxG cells (108CC15) [61] as well as isolated T cells and PMNs were washed in ice cold PBS and were centrifuged for 10 min at 250× *g*. The pellet was resuspended in RIPA lysis buffer (Santa Cruz Biotechnology, Dallas, TX, USA) and was incubated on ice for 30 min. After homogenizing the cells, the lysate was centrifuged for 20 min at 12,000× *g* and 4 °C. Protein concentrations were further determined by a BCA Assay (Pierce™ BCA Protein Assay Kit, Thermo Fisher Scientific, Waltham, MA, USA).

For SDS-Page, 5 µg protein was mixed 1:1 with Laemmli sample buffer (Bio-Rad Laboratories, Hercules, CA, USA), containing 2-mercaptoethanol and was loaded on a 10% denaturing gel (10% Mini-PROTEAN^®^ TGX Stain-Free™ Protein Gel (Bio-Rad Laboratories, Hercules, CA, USA).

The gel was blotted on a Trans-Blot Turbo Mini 0.2 µm PVDF membrane in the Trans-Blot Turbo system (Bio-Rad Laboratories, Hercules, CA, USA), and the membrane was consequently blocked for 1 h in TBST + 5% BSA. Incubation of the first antibody was carried out overnight at 4 °C. Based on the different proteins, different antibodies (diluted in blocking solution) were used (Table S3). After washing in TBST, the membrane was incubated with the secondary antibody for 1 h at room temperature. Protein bands were detected with Clarity Western ECL Substrate in a ChemiDoc imaging system (Bio-Rad Laboratories, Hercules, CA, USA).

Co-IP was performed using the Dynabeads™ Co-Immunoprecipitation Kit (Invitrogen, Waltham, MA, USA), following the manufacturer’s instructions. Ten micrograms/sample of anti-TAS1R3 or anti-mGlu_2_ antibody was covalently cross-linked to Dynabeads. The cell lysis and SDS-page procedures as well as the antibody concentrations were the same as described above for Western blot, using 12,000× *g* or 15,000× *g* for a 20 min centrifugation after cell lysis, to remove the membrane and any unsolubilized receptors [60].

### 4.9. Molecular Cloning

The protein-coding regions for human taste receptors *TAS1R1*, *TAS1R2*, and *TAS1R3* (NCBI reference sequences: NM_138697.4, NM_152232.5, NM_152228.2) were amplified using a polymerase chain reaction (PCR) from genomic DNA with gene specific primers (Table S4) and cloned EcoR1/Not1 into the expression plasmid pFN210A (#pFN210A SS-HaloTag^®^ CMV-neo Flexi^®^-Vector, Promega, Madison, WI, USA). The protein coding region of human *GRM2* (NCBI reference sequence: NM_000839.5) was obtained using gene synthesis from Genscript (Genscript Biotech, Piscataway, NJ, USA) in the appropriate expression plasmid pFN210A. Thus, coding regions of all receptors were cloned into plasmid pFN210A in such way that the recombinant receptors carried an N-terminal IL-6-HaloTag [64,65]. Using heat shock, competent *Escherichia coli* XL-1-blue cells (Agilent Technologies, Santa Clara, CA, USA) were transformed with plasmid-DNA, which was purified using the pure yield plasmid midiprep kit (Promega, Madison, WI, USA). Concentration of plasmid-DNA was determined using a NanoDrop™ One spectrophotometer (Thermo Fisher Scientific, Waltham, MA, USA) and adjusted to 250 ng/µL. The sequences were verified by Sanger sequencing (Eurofins Genomics, Ebersberg, Germany) using standardized vector internal primers (Table S5).

### 4.10. Cell Culture and Transient DNA Transfection

Human embryonic kidney (HEK-293) cells [103] were cultivated in 4.5 g/L D-glucose containing DMEM with 10% fetal calf serum, 2 mM L-glutamine, 100 units/mL penicillin, and 100 units/mL streptomycin at 37 °C and 5% CO_2_. One day before transfection, HEK-293 cells were cultured in 96-well plates (Nunclon™ Delta Surface, Thermo Fisher Scientific, Waltham, MA, USA) with a density of 12,000 cells per well. Using the ViaFect™ Transfection Reagent (Promega, Madison, WI, USA), 100 ng of taste receptor plasmid DNA (50 ng each for cotransfection), 50 ng of Gα_i_ protein subunit, and 50 ng of genetically modified luciferase pGloSensor^TM^-22FcAMP [63] (Promega, Madison, WI, USA) were transfected. For the analysis of the homodimers mGlu_2_ and TAS1R3, 50 ng of each receptor were used, together with 50 ng of mock. As a negative control, the vector-plasmid pFN210A without any taste receptor coding region was used (mock). A change in media took place on day three by removing the old and adding 100 µL new DMEM and, in the case of PTX stimulation experiments, DMEM with 0.5 µg/mL PTX. For transfecting NxG cells as a positive control for Western blot analysis, 1 million cells were seeded in a 15 cm dish in 9 mL DMEM. After one day of incubation, the cells were transfected with 10 µg receptor DNA and were harvested 42 h post transfection. 

### 4.11. cAMP Luminescence Assay

The cAMP luminescence assay was performed 40–42 h post transfection as reported previously [104]. The cells were incubated in a physiological salt buffer (pH 7.5) containing 140 mmol/L NaCl, 10 mmol/L HEPES, 5 mmol/L KCl, 1 mmol/L CaCl_2_, 10 mmol/L D-glucose, 0.1% DMSO, and 2% of beetle luciferin sodium salt. After incubation for 50 min in the dark, the basal luminescence signal was measured in triplicates in a GloMax^®^ Discover Microplate Reader (Promega, Madison, WI, USA). Ligand stock solutions were prepared in water and were diluted in salt buffer containing 7.5 µM forskolin. The final forskolin concentration was 2.5 µM, and the final DMSO concentration on the cells was 0.1%. After ligand application, the luminescence signals for each well were measured for 21 min, until a signal plateau was reached.

### 4.12. Data Analysis of cAMP Luminescence Assay

The raw luminescence data of cAMP luminescence measurements were obtained as excel sheets from the GloMax^®^ Discover Microplate Reader. For each well, three data points before (basal luminescence) and two after ligand stimulation (highest signals at plateau) were averaged, and the corresponding baseline was subtracted from each signal. For concentration–response relations, measurements were performed from three independent transfection experiments in triplicates. The baseline-corrected data were then normalized to the minimum ligand concentration. By fitting the function $f\left(x\right)=min+\left(max-min\right)/(1+(x/{IC}_{50}{)}^{-hillslope})$ to the data by nonlinear regression (SigmaPlot 14.0, Systat Software), IC_50_ values and curves were derived. The data are presented as mean ± SD.

### 4.13. ELISA

For ELISA measurements, PMN cells were pre-stimulated for 2 h with 50 µM MSG in combination with 40 nM mGlu_2_ antagonist 1 and/or 0.5 µM Lactisole, following a 4 h incubation step with fMLF (0.01–10 nM). The final concentration of mGlu_2_ receptor agonist LY379268 was 20 nM and of mGluR2 antagonist 1300 nM. Cell supernatants were collected, and ELISA assays for IL-8 were performed, according to the manufacturer’s recommendations (R&D Systems, Minneapolis, MN, USA). OD signals were detected in a M200 infinite plate reader (Tecan Group, Männedorf, Switzerland) at wavelengths of 450 and 540 nm, with subsequent wavelength correction by subtracting the OD_540_ values from the OD_450_ values. A standard curve was generated by plotting the mean absorbance of the IL-8 standard (x-axis) against the respective target concentrations (y-axis), and the IL-8 concentrations were determined using regression analysis based on the standard curve. For every treatment, cells were measured in duplicates and were normalized to negative control (medium treatment). The detection limit for IL-8 was 31.3–2000 pg/mL.

### 4.14. Intracellular Ca^2+^ Fluorescence Measured Using Laser-Guided Flow Cytometry

Ca^2+^-fluorescence was measured as recently reported [31]. Briefly, 0.5 Mio PMNs/mL were centrifuged at 300× *g* for 10 min, following a resuspension of the cell pellet in 1 mL calcium buffer, containing final concentrations of 1 mM probenecid, 0.04% Pluronic^®^ F-127, and 4 µM of Ca^2+^-sensitive fluophor Fluo-4 AM. After incubation for 45 min at 37 °C and 5% CO_2_, the cell suspension was washed twice with calcium buffer and centrifuged at 300× *g* for 10 min with a final resuspension of the cell pellet in 1 mL calcium buffer without Fluo-4. PMNs were stimulated with 50 µM MSG or 20 nM LY379268, with or without 100 nM mGluR2 antagonist 1 diluted in the same calcium buffer and incubated for 2 h at 37 °C and 5% CO_2_. Following a centrifugation at 300× *g* for 10 min, the cell pellet was resuspended in 500 µL MACSQuant^®^ Running Buffer (Miltenyi Biotec, Bergisch Gladbach, Germany). Using a laser-guided flow cytometry, i.e., MACSQuant^®^ Analyzer 16 (Miltenyi Biotec, Bergisch Gladbach, Germany), the fluorescence was measured until 10,000 events were detected. For excitation and emission, wavelengths were 494 nm and 506, respectively. Data were analyzed using SigmaPlot 14.0 (Systat Software GmbH distributed from Inpixon GmbH, Düsseldorf, Germany). Solvent control (0.1% DMSO) was subtracted from each measurement.

### 4.15. Statistical Analysis

Normality testing (Shapiro–Wilk), Student’s *t*-test, or Wilcoxon signed rank test for the in vitro data was performed with SigmaPlot 14.0 (Systat Software GmbH distributed from Inpixon GmbH, Düsseldorf, Germany). The individual figures were generated with SigmaPlot 14.0 and OriginPro 10.0 (OriginLab Corporation, Northampton, MA, USA).

## Figures and Tables

**Table 1:** IC_50_ values of MSG for mGlu_2_, TAS1R3, and their heterodimer.

G-Protein	mGlu_2_	mGlu_2_/TAS1R3	TAS1R3
G_αGustducin_	56.14 ± 10.12 ^a^	25.66 ± 6.26 ^A^*	n.d.
G_αi1_	18.11 ± 2.91 ^a^	5.88 ± 0.97 ^B^*	n.d.
G_αi2_	19.88 ± 2.46 ^a^	7.79 ± 0.52 ^A^*	n.d.
G_αi3_	8.38 ± 0.50 ^b^	4.97 ± 0.59 ^B^*	n.d.

Values are given as mean ± SD (*n* = 3–4) in µmol/L (µM). Significance of difference between mGlu_2_ and mGlu_2_/TAS1R3 was tested using the two-sided student’s *t*-test: (*) *p* ≤ 0.05. Different letters indicate significant differences (*p* ≤ 0.05) between G_αi3_ and the other G-protein subunits. n.d., not determined.

**Table 2:** IC_50_ values of MSG for mGlu_2_ and in combination with TAS1Rs.

Receptors	IC_50_ Value
mGlu_2_	8.38 ± 0.50 ^a^
mGlu_2_/TAS1R3	4.97 ± 0.59 ^b^
mGlu_2_/TAS1R2	10.84 ± 2.13 ^a^
mGlu_2_/TAS1R1	10.62 ± 1.26 ^a^

Values are given as mean ± SD (*n* = 3–4) in µmol/L (µM). Different letters indicate significant differences (*p* ≤ 0.05, two-sided student’s *t*-test).

## Data Availability

The data presented in this study are available on request from the corresponding author.

## Supplementary Materials

The following supporting information can be downloaded at: https://www.mdpi.com/article/10.3390/ijms241612942/s1.
