# Supplementary material for: Heterodimerization of Chemoreceptors TAS1R3 and mGlu2 in Human Blood Leukocytes
Source: Int J Mol Sci. 2023 Aug 18;24(16):12942. doi: 10.3390/ijms241612942 (PMC10454557; doi:10.3390/ijms241612942)
Supplement: Supplementary file 1 [file ijms-24-12942-s001.zip › ijms-2481134-supplementary.pdf]

Heterodimerization of chemoreceptors TAS1R3 and $mGlu_2$ in human blood leukocytes

Lena Ball[1;2], Julia Bauer[2] and Dietmar Krautwurst[2*]

[1] TUM School of Life Sciences, Technical University of Munich, 85354 Freising, Germany

[2] Leibniz-Institute for Food Systems Biology at the Technical University of Munich, Lise-Meitner-Str. 34, 85354 Freising, Germany

[*] Correspondence: d.krautwurst.leibniz-lsb@tum.de

Contents:

Table S1: Oligonucleotide primer sequences for RT-qPCR..2

Table S2: Oligonucleotide primer and probe sequences for ddPCR..2

Table S3: Antibodies for Western Blot and Co-IP..3

Table S4: Oligonucleotide primer sequences for molecular cloning 3

Table S5: Vector-internal oligonucleotide primer sequences..3

Table S6: EC_{50} values of fMLF for treated and untreated PMNs..3

Figure S1: Gene expression analysis of *GRM2* isoforms in PMNs and T-cells..........................4

Figure S2: Protein expression of $mGlu_2$ and TAS1R3 in human T-cells....................................4

Figure S3: Specificity of the HaloTag-TAS1R3 : NanoLuc-mGlu2 BRET signal by titration of the HaloTag BRET acceptor...5

Figure S4: MSG did not affect homo- or heteromeric BRET signals of TAS1R3 and mGlu2....5

Figure S5: Cell-surface expression of $mGlu_2$ and TAS1R3..6

Figure S6: mGlu2 and TAS1R3 receptor antagonists have no effect on fMLF-induced IL-8 secretion in isolated PMNs...7

Figure S7: The $mGlu_2$-receptor agonist LY379268 facilitated a fMLF-induced IL-8 secretion, which was prevented by the mGluR2 antagonist 1...7

References 8

Table S1: Oligonucleotide primer sequences for RT-qPCR

Gene	Oligo-nucleotide	T_M (°C)		Sequence 5´→ 3´
GAPDH	dk-918a	58	fw	CAT GGG TGT GA ACCA TGA GA AGTA TGA C
	dk-919	58	rv	CAC GGA AGG CCA TGC CAG TGA GCT TC
ACTB	ab-270	58	fw	GCC AAC CGC GAG AAG ATG ACC
	ab-271	58	rv	CAG GTC CAG ACG CAG GAT GG
GRM1	ab-124	58	fw	GGA GGA AAA GCC TGG GAG TGG
	ab-125	58	rv	GGC TGT TCC TTG GCA AGC ATG G
GRM2	ab-126	58	fw	GGA GGG AGA CTT GGT GCT GG
	ab-127	58	rv	GGT GCG GGT CAC GGT TGA TG
GRM3	ab-128a	59	fw	GCG CTT CTT CAA CTG GAC CTA CG
	ab-129a	58	rv	GGA CTT GCG GAT GTT GGA GCG
GRM4	lb-027	58	fw	GCG CAA CGA TTC TGC CGA GTA C
	lb-028	58	rv	GTA CCC TGT GCA AGG CTC GC
GRM5	ab-132	58	fw	GGC GGT CCG TGA ACA GTA TGG
	ab-133	58	rv	GGC CCA ATG ACC CCT ACT ATG G
GRM6	ab-134a	58	fw	GGC GAT GGA ACC CAC TGA TGG
	ab-135a	58	rv	CCA GTC TGA GGG TCT CTG CC
GRM7	ab-136	58	fw	GCA CTC AAT CCG GAT CGA GGG
	ab-137	58	rv	GCG CCT GGA CGA AAG TAA GCG
GRM8	lb-029	58	fw	GAT CTC GAG GGA GAT TGG TGG TG
	lb-030	58	rv	CCT CCT GAT GTC ATC CTC ATT GGC
TAS1R1	ab-114	58	fw	GGT GGA CCT GGA TCT CTC TGG
	ab-115a	58	rv	GAG GCA CTG CAT CCT CTC ATC G
TAS1R2	ab-102	58	fw	GCT GGA ACT GGA TCA TTG TGC TGG
	ab-103	58	rv	GTG CTC TGC TGC AGC TTG TCC
TAS1R3	ab-106	58	fw	GGT GCT AGC ATG GAG CTG CTG
	ab-107	58	rv	GAG AAG ATG CTC AGG CCC TGC

T_M = melting temperature, fw = forward, rv = reverse. Size of amplicons: 150 - 308 bp

Table S2: Oligonucleotide primer and probe sequences for ddPCR

GRM2 **Isoform (sequence accession number)**	T_M (°C)		**Sequence (shown are always 1. forward primer, 2. reverse primer, 3. probe)**
Isoform 1 (NM_001349116.2)	58	fw	CGC TCC ACT CCG ATT CTC
	58	rv	CAG TCT CGC TGC CGG AA
	68	probe	[FAM] CGC GCC AGA GCC TGG ACC CT [BHQ1]
Isoform 2 (NM_001349117.2)	58	fw	ACC TGA AAG TCT CCT GAA ATC
	58	rv	CAA GCT TTG TCT CCC ACT GT
	68	probe	[FAM] AGT GCC AGC TTT AAG AGA AAT GAC TGC [BHQ1]
Isoform 3 (all isoforms including NM_000839.5)	58	fw	TCT GCT ACT GCA TGA CCT TCA
	58	rv	CAA TGC GGT TGG TCT TGG T
	68	probe	[FAM] CCA CGG CAG TGT GTA CCT TAC GG [BHQ1]

T_M = melting temperature, fw = forward, rv = reverse. Size of amplicons: 126 - 131 bp

Table S3: Antibodies for Western Blot and Co-IP

Target	Antibody	Dilution
TAS1R3	Anti-TAS1R3 Rabbit antibody (#A100296) (antibodies.com, Cambridge, United Kingdom)	1:1000
mGlu2	mGluR2 Rabbit pAb (#A10561) (ABclonal Inc., Woburn, USA)	1:1000
Secondary antibody	Goat anti-Rabbit IgG (H+L) Cross-Adsorbed Secondary Antibody, HRP, Invitrogen (#G-21234) (Invitrogen, Waltham, USA)	1:2000

Table S4: Oligonucleotide primer sequences for molecular cloning

Gene	Oligo-nucleotide	T_M (°C)		Sequence 5'→ 3'
TAS1R1	309	62	fw	CTGC *GAATTC* **ATG** CTG CTC TGC ACG GCT CGC C
	310	62	rv	CTGC *GCGGCCGC* TCA GGT GGA GCC GCA GCG CC
TAS1R2	311	62.5	fw	CTGC *GAATTC* **ATG** GGG CCC AGG GCA AAG ACC ATC
	312	62	rv	CTGC *GCGGCCGC* CTA GTC CCT CCT CAT GGT GTA GCC C
TAS1R3	313	62	fw	GTGC *GAATTC* **ATG** CTG GGC CCT GCT GTC CTG G
	314	62	rv	CTGC *GCGGCCGC* TCA CTC ATG TTT CCC CTG ATT TCC TGT GTT C

T_M = melting temperature, fw = forward, rv = reverse. Italic letters highlight the restriction sites. Start codon is bold.

Table S5: Vector-internal oligonucleotide primer sequences

Vector	Oligo-nucleotide	T_M (°C)		Sequence 5'→ 3'
pFN210A	520	60	fw	GTG GAC ATC GGC CCG GGT C
	550	52	rv	CAC AAA TAA AGC ATT TTT TTC ACT GC

T_M = melting temperature, fw = forward, rv = reverse

Table S6: EC_{50} values of fMLF for treated and untreated PMNs

Treatment	EC_{50} value
Untreated	2.38 ± 1.09
MSG	2.15 ± 1.11
mGluR2 antagonist 1	1.19 ± 0.28
Lactisole	2.10 ± 1.13
mGluR2 antagonist 1 + Lactisole	1.34 ± 0.26

Values are given as mean ± SD (*n*= 6 - 13) in µmol/L (µM)

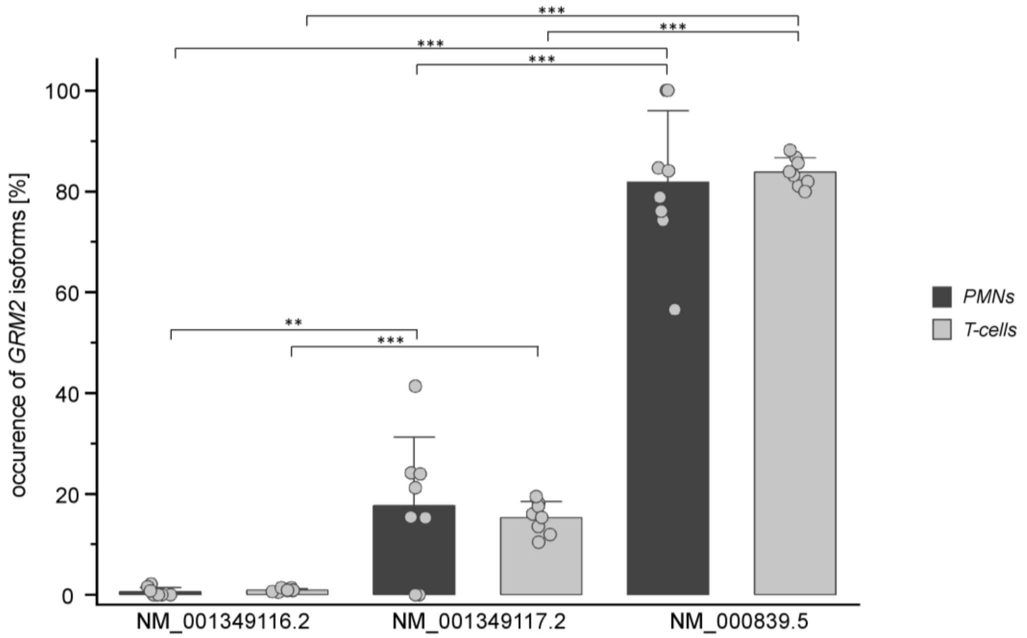

Figure S1: Gene expression analysis of *GRM2* isoforms in PMNs and T-cells

Droplet digital-PCR analysis of *GRM2* isoforms in PMNs (dark grey) and T-cells (grey). Data are shown as mean ± SD in n=8 different blood samples. Significance of difference between isoforms was tested by two-sided paired student's t-test: (∗∗∗) p ≤ 0.001; (∗∗) p ≤ 0.01.

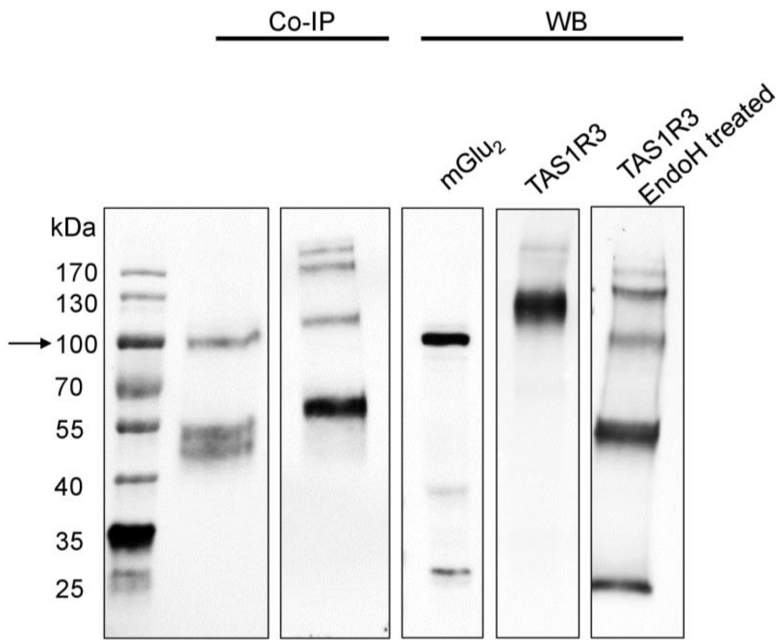

Figure S2: Protein expression of $mGlu_2$ and TAS1R3 in human T-cells

For Co-IP assay, cell lysates from T-cells were incubated with anti-TAS1R3 (lane 1,) or anti-$mGlu_2$ antibody (lane 2), attached to Dynabeads®. For Western Blot, whole cell lysates and immunoprecipitates were subjected to SDS-PAGE and were analyzed by immunoblotting using anti-$mGlu_2$ (lanes 1+3) or anti-TAS1R3 antibodies (lanes 2+4). The predicted size of $mGlu_2$ protein is ∼110 kDa, for TAS1R3 protein ∼ 97 kDa, indicated by the black arrow. Lane 5: a ~97 kDa band was obtained with EndoH treated T-cell lysates.

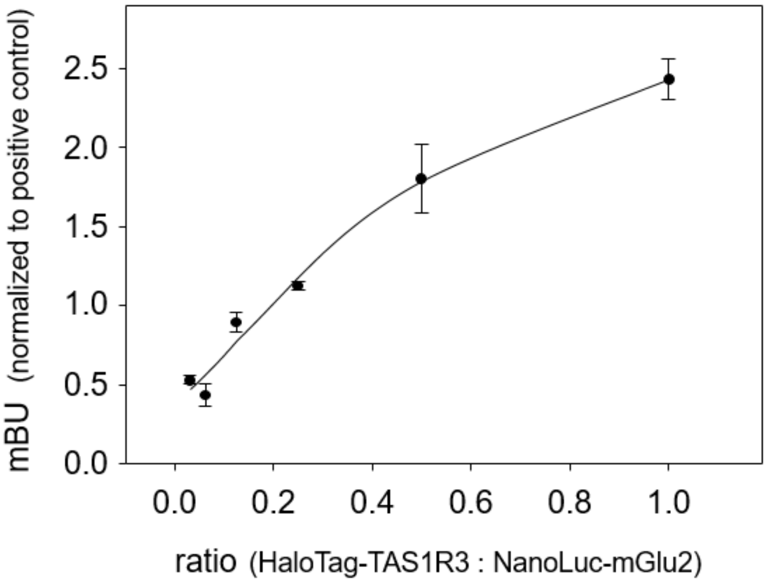

Figure S3: Specificity of the HaloTag-TAS1R3 : NanoLuc-mGlu2 BRET signal by titration of the HaloTag BRET acceptor. The resulting hyperbolic saturation curve suggests specificity of the BRET signal, when titrating HaloTag-TAS1R3, but keeping NanoLuc-mGlu2 (donor) constant: a ratio of ‘1‘ depicts 50 ng plasmid DNA/well each, for donor and acceptor. Receptor plasmids were transfected into HEK-293 cells using Fugene® HD (Promega, Walldorf, Germany). Data are presented as the means ± SD of n=2 independent experiments. mBU, milliBRET units, normalized to positive control (PPI p53-pFN : MDM2-NL).

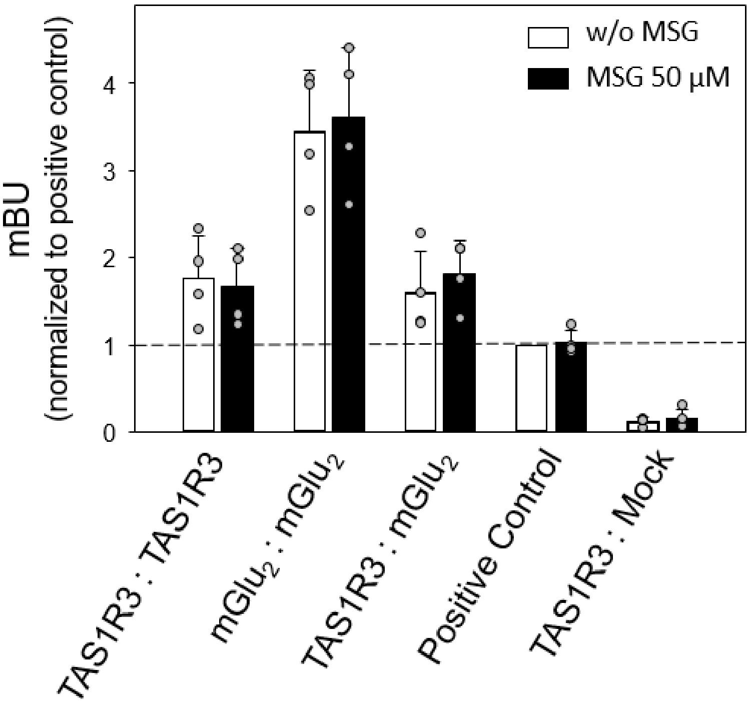

Figure S4: MSG did not affect homo- or heteromeric BRET signals of TAS1R3 and mGlu2. The first mentioned receptor was always expressed out of vector pFN210A, carrying the IL-6-HaloTag (BRET acceptor), the second one out of pNsecNLuc, carrying the NanoLuc luciferase (BRET donor). Receptor plasmids were transfected into HEK-293 cells using Fugene® HD (Promega, Walldorf, Germany). Data are presented as the means ± SD of n=4 independent experiments, normalized to positive control (PPI p53-pFN : MDM2-NL) in the absence of MSG (dashed line). mBU, milli-BRET units.

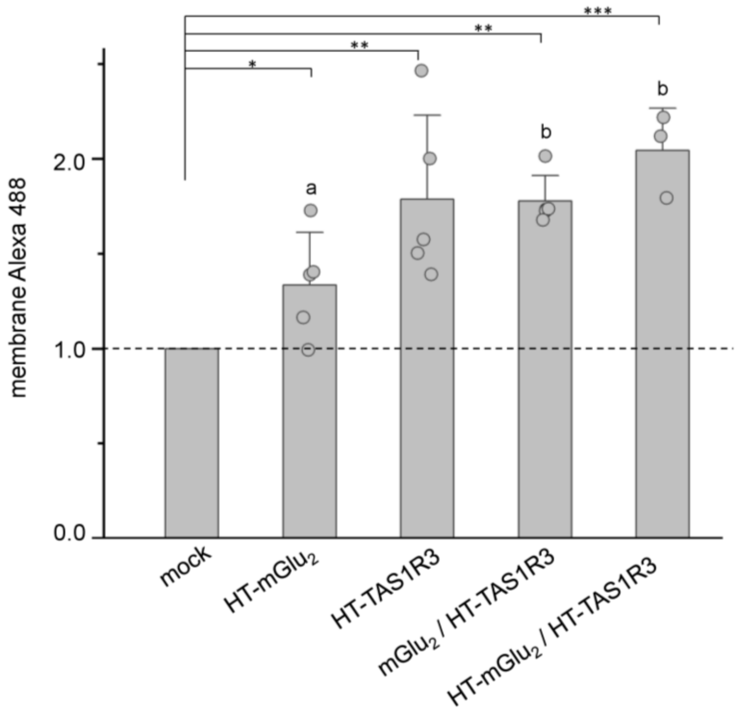

Figure S5: Cell-surface expression of $mGlu_2$ and TAS1R3.

Flow cytometry analysis showing the relative surface expression of $mGlu_2$, TAS1R3 and its heterodimer. Data are shown as mean ± SD (n = 3 – 5). . Surface expression over mock (dashed line) of tagged and untagged receptors was significant as tested by two-sided student's t-test: (∗∗∗) $p \leq 0.001$; (∗∗) $p \leq 0.01$; (∗) $p \leq 0.05$. Different letters indicate significant differences ($p \leq 0.05$) between HT-$mGlu_2$ and $mGlu_2$ / HT-TAS1R3 or HT-$mGlu_2$ / HT-TAS1R3.

Flow cytometry

For measuring the surface expression of $mGlu_2$ and TAS1R3, HEK293 cells were cultivated with a density of 96,000 cells per well in a 12-well plate. The transfection on the next day was performed by transfecting 800 ng of receptor (400 ng each for cotransfection), 400 ng of $G_{\alpha i3}$ protein subunit and 400 ng of genetically modified luciferase pGloSensor^{TM}-22FcAMP [1] (Promega, Madison, USA) using the ViaFect™ Transfection Reagent (Promega, Madison, USA), as described earlier [2].

The receptors $mGlu_2$ and TAS1R3 were each transfected with 400 ng mock to obtain the same amount of DNA as in the co-transfected cells. 42 h post transfection, the cells were stained with the cell-inpermeant HaloTag® Alexa Fluor® 488 Ligand (ex/em= 499/518 nm) and were incubated at 37 °C and 5 % CO2 for 30 min in a cell culture incubator. After washing the cells twice with serum free media the receptor surface expression of 10,000 cells each was analysed in the flow cytometer MACSQuant Analyzer (Miltenyi Biotec, Bergisch Gladbach, Germany). To exclude dead cells a forward (FSC: 295 V) - and sidescatter (SSC: 592 V) gate was set and the FITC signal (B1-channel; HaloTag® Alexa Fluor® 488 Ligand) was detected with 370 V. The data were analysed using the FlowLogic software (Version 7.3) (Inivai Technologies, Mentone VIC, Australia).

References

1. Binkowski, B.; Fan, F.; Wood, K. Engineered luciferases for molecular sensing in living cells. *Current opinion in biotechnology* **2009**, *20*, 14-18, doi:10.1016/j.copbio.2009.02.013.
2. Noe, F.; Geithe, C.; Fiedler, J.; Krautwurst, D. A bi-functional IL-6-HaloTag® as a tool to measure the cell-surface expression of recombinant odorant receptors and to facilitate their activity quantification. *Journal of Biological Methods* **2017**, *4*, doi:10.14440/jbm.2017.207.

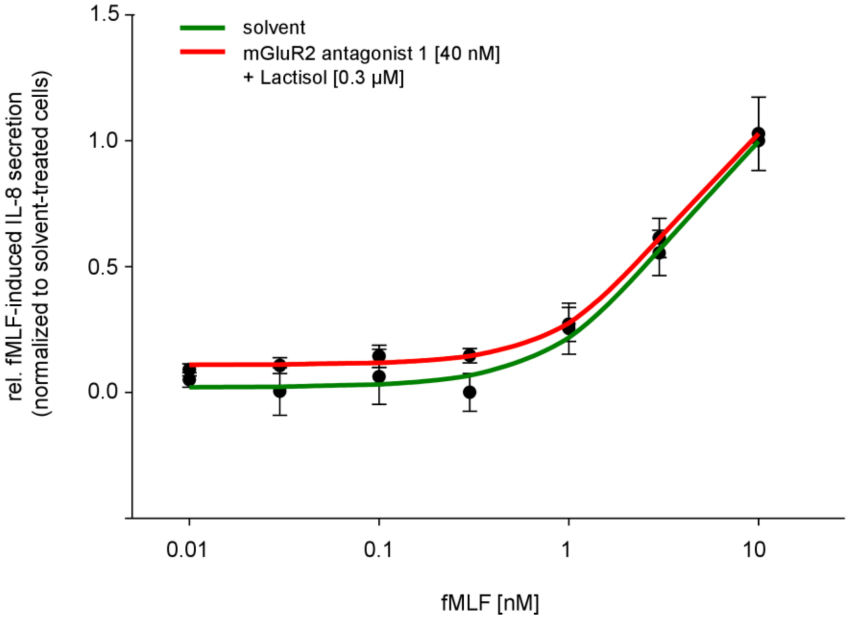

Figure S6: Receptor antagonists have no effect on fMLF-induced IL-8 secretion in isolated PMNs

4 h fMLF-induced IL-8 secretion in isolated PMNs in the absence of MSG but in presence of mGlu2-selective antagonist-1 and TAS1R3-specific antagonist Lactisole. Changes of IL-8 concentration are normalized to RPMI-treated samples without antagonists ("solvent"). Data are the mean ± SEM (n=4).

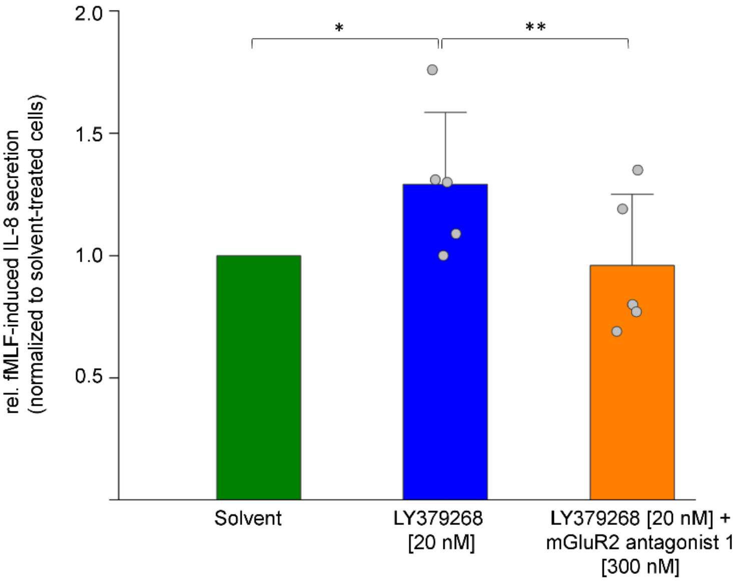

Figure S7: The $mGlu_2$-receptor agonist LY379268 facilitated a fMLF-induced IL-8 secretion, which was prevented by the mGluR2 antagonist 1.

4 h fMLF-induced (3 nM) IL-8 secretion in isolated PMNs, after 2 h pre-stimulation with 20 nM $mGlu_2$-receptor agonist LY379268 in the absence or presence of mGluR2 antagonist 1. Changes of IL-8 concentration are normalized to RPMI-treated samples without LY379268 ("solvent"). Data are the mean ± SEM (n=5). Significance of difference was tested by one-sided student's t-test: (**) $p \leq 0.01$; (*) $p \leq 0.05$.
